# Developing a hypertension visualization risk prediction system utilizing machine learning and health check-up data

**DOI:** 10.1038/s41598-023-46281-y

**Published:** 2023-11-02

**Authors:** Jinsong Du, Xiao Chang, Chunhong Ye, Yijun Zeng, Sijia Yang, Shan Wu, Li Li

**Affiliations:** 1 School of Public Health and Clinical Medicine, Hangzhou Normal University, Hangzhou, 311121 China; 2 Preventive Treatment of Disease and Health Management Center, The Affiliated Hospital of Hangzhou Normal University, Hangzhou, 311121 China

**Keywords:** Endocrinology, Health care, Risk factors, Signs and symptoms

## Abstract

As an important risk factor for many cardiovascular diseases, hypertension requires convenient and reliable methods for prevention and intervention. This study designed a visualization risk prediction system based on Machine Learning and SHAP as an auxiliary tool for personalized health management of hypertension. We used ten Machine Learning algorithms such as random forests and 1617 anonymized health check data to build ten hypertension risk prediction models. The model performance was evaluated through indicators such as accuracy, F1-score, and ROC curve. We used the best-performing model combined with the SHAP algorithm for feature importance analysis and built a visualization risk prediction system on the web page. The LightGMB model exhibited the best predictive performance, and age, alkaline phosphatase, and triglycerides were important features for predicting the risk of hypertension. Users can obtain their risk probability of hypertension and determine the focus of intervention through the visualization system built on the web page. Our research helps doctors and patients to develop personalized prevention and intervention programs for hypertension based on health check data, which has significant clinical and public health significance.

## Introduction

Hypertension as a common and serious health problem worldwide, is a crucial risk factor for cardiovascular diseases such as coronary heart disease and stroke^1–3^. In China, the population of hypertension patients has exceeded 200 million, and the number is growing at a rate of 10 million per year^4,5^. Early intervention and control can significantly reduce the incidence and mortality of hypertension and its complications^6^, so it is of great significance to establish a highly efficient and accurate risk prediction system for hypertension management.

Traditionally in medical practice, doctors usually evaluate the risk of a disease based on lifestyle and family history^7,8^. This approach is subject to subjective factors insufficient for the processing of large-scale complex medical data. By conducting in-depth analysis and modeling of medical data, potential risk factors for specific diseases can be identified and quantified, thus accurately and efficiently predicting the risk of related diseases^9–13^. In recent years, Machine Learning (ML) technology as a powerful data analysis tool has been demonstrating huge potential in disease risk prediction. Zhang et al. used six different ML algorithms to predict the risk of type 2 diabetes in rural populations in China^14^. Mohammed et al. used ML techniques to analyze medical records and MRI images for early diagnosis of Alzheimer's disease^15^. Yan et al. have constructed a prediction system for diagnosing coronary heart disease based on ML technology, which can minimize unnecessary invasive examinations^16^. It can be seen that ML technology can predict the risk of hypertension by learning the patterns and rules in medical data. However, although ML technology has achieved excellent performance in disease prediction, due to its "black box" characteristics, the internal operation mechanism of the model is not transparent and it is difficult to explain the prediction results, which undermines the trust of doctors and patients in the predictive results and limits its application in clinical practice^17,18^.

**Figure 1:**
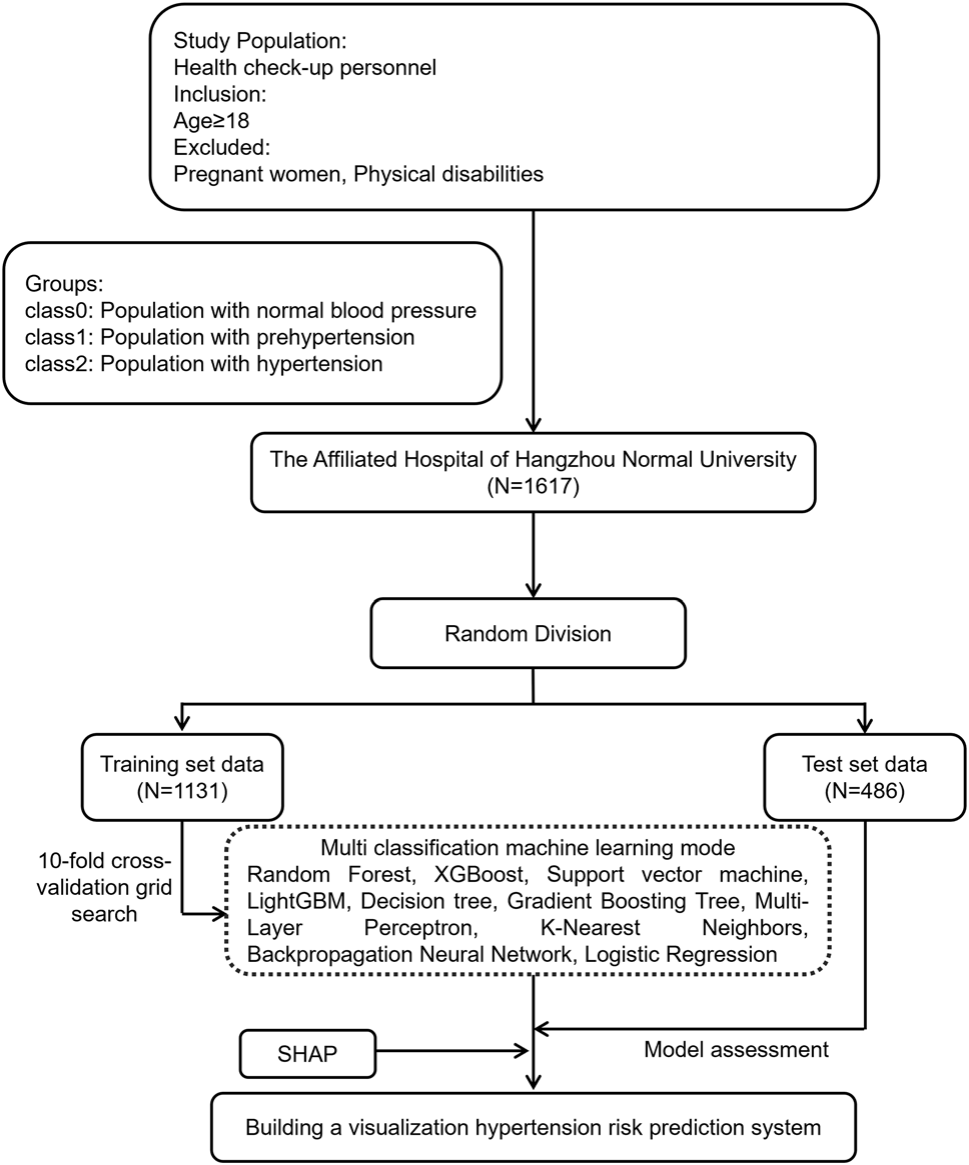
Flowchart describing the overall research framework.

To address this issue, this research developed a visualization risk prediction system for hypertension based on ML technology (Fig. 1). This system will use health check-up data in combination with ten different ML algorithms to accurately predict prehypertension and hypertension risk. At the same time, we use the Shapley Additive Explanation (SHAP) algorithm to interpret the predictive results with a visually intuitive interface to present the results and evidence of the prediction. This visualization risk prediction system is efficient, accurate, convenient and can provide doctors and patients with more comprehensive hypertension risk assessment and management services, and it has broad clinical applicational prospects.

## Methods

### Study population

This study is a retrospective research project that has obtained approval from the Ethics Committee of Hangzhou Normal University Affiliated Hospital (protocol code 2023E2-KS-110 and date of July 2023). Due to the research involving previously collected anonymous data and the absence of a direct risk of individual privacy disclosure, in accordance with Article 39 of the 'Ethical Review of Biomedical Research Involving Humans' in China, the Ethics Committee of Hangzhou Normal University Affiliated Hospital has waived the requirement for informed consent. Throughout the entire research process, we rigorously adhere to ethical principles, maintaining transparency and integrity, while conducting all experiments in accordance with relevant guidelines and regulations. We retrieved health check data from the Health Management Center of Hangzhou Normal University Affiliated Hospital from May 31, 2022, to May 31, 2023, including 18 years old and above and complete blood pressure data but excluding pregnant women and people with physical disabilities. A dataset was generated that includes 1617 health check records. According to the standard of the JNC-720, we divided the subjects in the dataset into normal blood pressure group (120 ≤ systolic blood pressure < 139 or 80 ≤ diastolic blood pressure < 89, denoted as class 1) and hypertension group (140 ≤ systolic blood pressure and 90 ≤ diastolic blood pressure, denoted as class 2).

### Data description and processing

The dataset includes the age, gender, lifestyle, blood routine, biochemical examination, etc. of the examined people, involving 69 variables (among them, 12 variables have a missing rate of more than 30% and 9 variables have a missing rate of less than 30%). To ensure data quality, the Multiple Imputation by Chained Equation (MICE) algorithm was used to fill variables with a missing rate of less than 30%^19,20^, and the fidelity of variables after filling was evaluated with mean, median, and standard deviation. Variables with a missing rate greater than 30% were deleted, finally retaining 57 feature variables. Descriptive statistics were carried out with SPSS 25^21^. Continuous data with a normal distribution are represented by the mean, and comparisons between groups were conducted by one-way analysis of variance. Non-normally distributed data are represented by the median, and comparisons between groups were made using rank-sum tests. Categorical variables are represented by counts, and chi-square tests were used for comparisons between groups.

### Machine learning models

This study uses Python 3.11 to design machine learning models, using the train_test_split algorithm to randomly divide the dataset into a training set (70%) and a test set (30%). This splitting method does not affect the class distribution in the dataset. The problem of unbalanced sample distribution in the training set was solved through SMOTE oversampling techniques^22^, and the model parameters were selected using 10-fold cross grid search. Models were built using several algorithms: Random Forest (RF)^23^, XGBoost (XGB)^24^, Support Vector Machine (SVM)^25^, LightGBM (LGB)^26^, Decision Tree (DT)^27^, Gradient Boosting Tree (GBT)^28^, Multilayer Perceptron (MLP)^29^, K-Nearest Neighbors (KNN)^30^, Back Propagation Neural Network (BPNN)^31^, and Logistic Regression (LR)^32^. Among them, RF, XGB, LGB, and GBT are ensemble learning algorithms, and the models constructed generally have superior performance; models constructed by SVM, MLP, and BPNN algorithms are good at handling non-linear data; models designed by LR, DT, and KNN are simple and have strong interpretability. In this research, the performance of models built using the aforementioned ten algorithms was assessed and the best performing model was chosen as the hypertension risk prediction model.

### Risk prediction system

We used the following six indicators to evaluate the classification performance of ML models on the test set: Accuracy (Formula S1), Precision (Formula S2), Recall (Formula S3), F1-score (Formula S4), macro-average ROC curve, and micro-average ROC curve^33^. We chose the ML model with the strongest overall performance as the best model, combined with the Shapley Additive Explanations (SHAP) to construct an online prediction system^34^.

## Results

### Research subjects

In this study, we used MICE to fill in the missing values of nine feature variables. After filling, there are no significant changes in these variables' mean, median, and standard deviation, demonstrating that the input data are good at preserving the integrity of the original data (Table S1). The dataset contains 1617 anonymized health check records. Table S2 shows the distribution of variables. The dataset contains 867 people (53.62%) with normal blood pressure, 557 people (34.45%) with prehypertension, and 193 people (11.93%) with hypertension. Similar to the results of this study, Wang et al. compiled 47 studies, showing that the prevalence of hypertension among Chinese adults is about 9.1% (95% confidence interval 4.1–14.1)^35^. This suggests that our data has good accuracy and reliability. There are differences among groups for 30 feature variables such as age, gender, drinking, pulse, weight, HbA1c, and alkaline phosphatase (ALP).

### Classification performance

**Figure 2:**
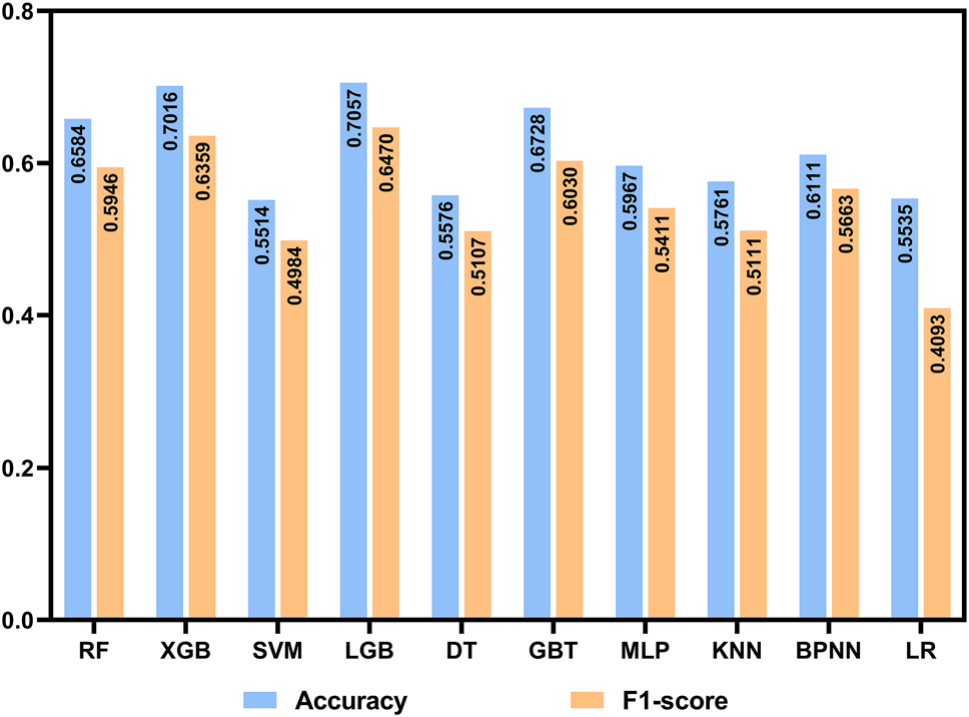
The accuracy and average F1-score of ML models. The blue bar represents accuracy, and the yellow bar represents the average F1-score.

**Figure 3:**
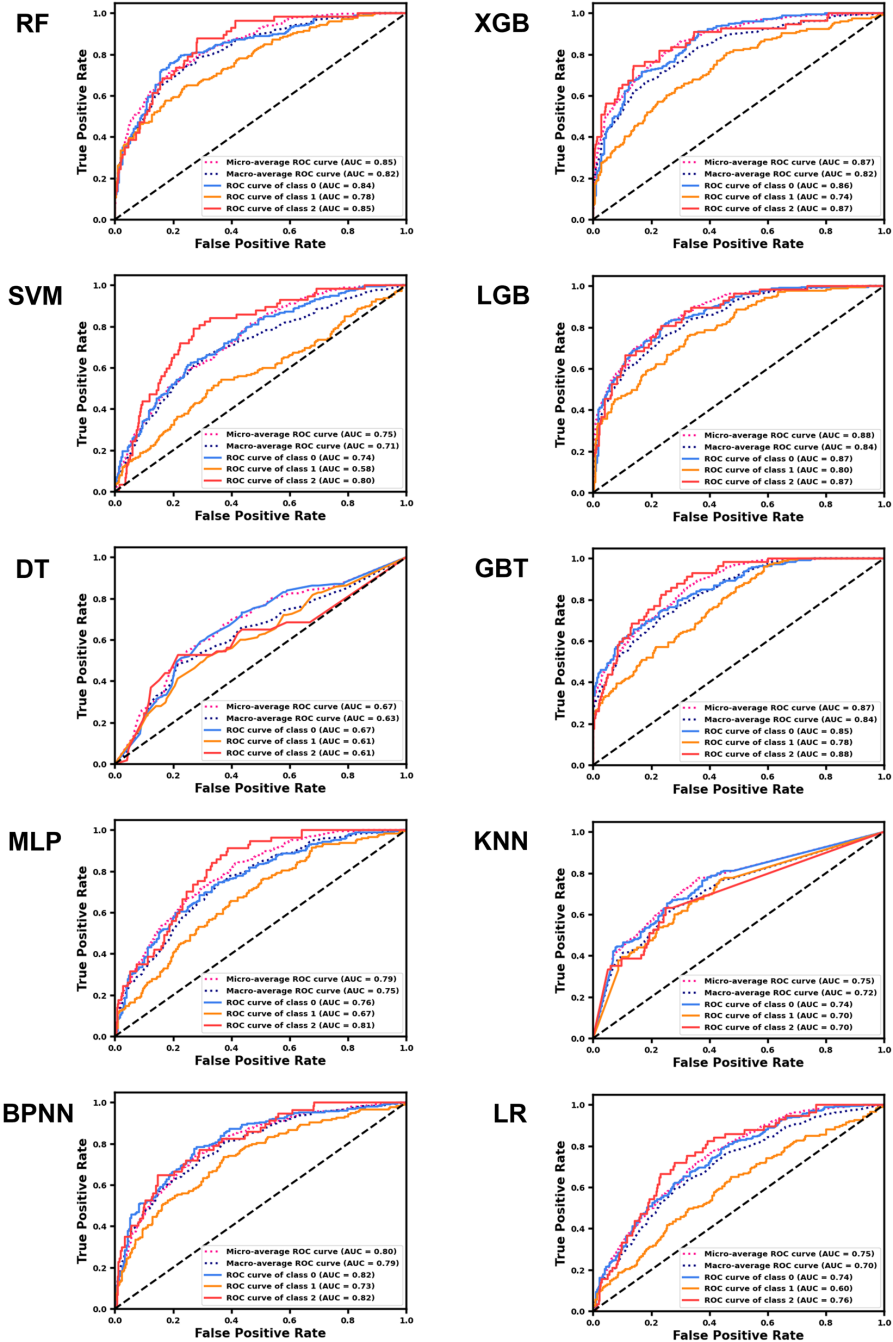
ROC curve of ML models. Normal blood pressure (class 0) is represented by the blue line, prehypertension (class 1) is represented by the yellow line, and hypertension (class 2) is represented by the red line. The micro-average curve is obtained by weighing all sample true positives and false positives, represented by the red dashed line. The macro-average curve is obtained by averaging the ROC curves of the three categories, which is represented by the blue dashed line.

Accuracy is a common index for assessing the performance of ML models which refers to the proportion of correct predictions made by the model to the total number. The F1-score is the harmonic mean of precision and recall, and it can better measure the quality and adaptability of the model when dealing with unbalanced data^36^. As shown in Fig. 2 and Table S3, the accuracy of the LGB and XGB models is higher than other models, but there is no significant difference between them (0.7057 vs 0.7016). The average F1-score of the LGB model is higher than XGB (0.6470 vs 0.6359), indicating that it can better balance precision and recall (Table S2). Besides, the ROC curve is also an index to judge the classification ability of the model. The larger the area under its curve (AUC), the better the model's classification performance. The macro-average ROC curve is the average of the ROC curves for each class, while the micro-average ROC curve is drawn based on the true positives and false positives of all samples. The former aims to highlight the performance of each category, while the latter emphasizes the overall performance. As shown in Fig. 3, the AUC values of the macro-average ROC curve and micro-average ROC curve of the LGB model are 0.84 and 0.88 respectively, which are higher than other models. Taking all indicators into account, we conclude that LGB (detailed parameters can be found in the Supplementary) is the best model for predicting the risk of hypertension based on health check data.

**Figure 4:**
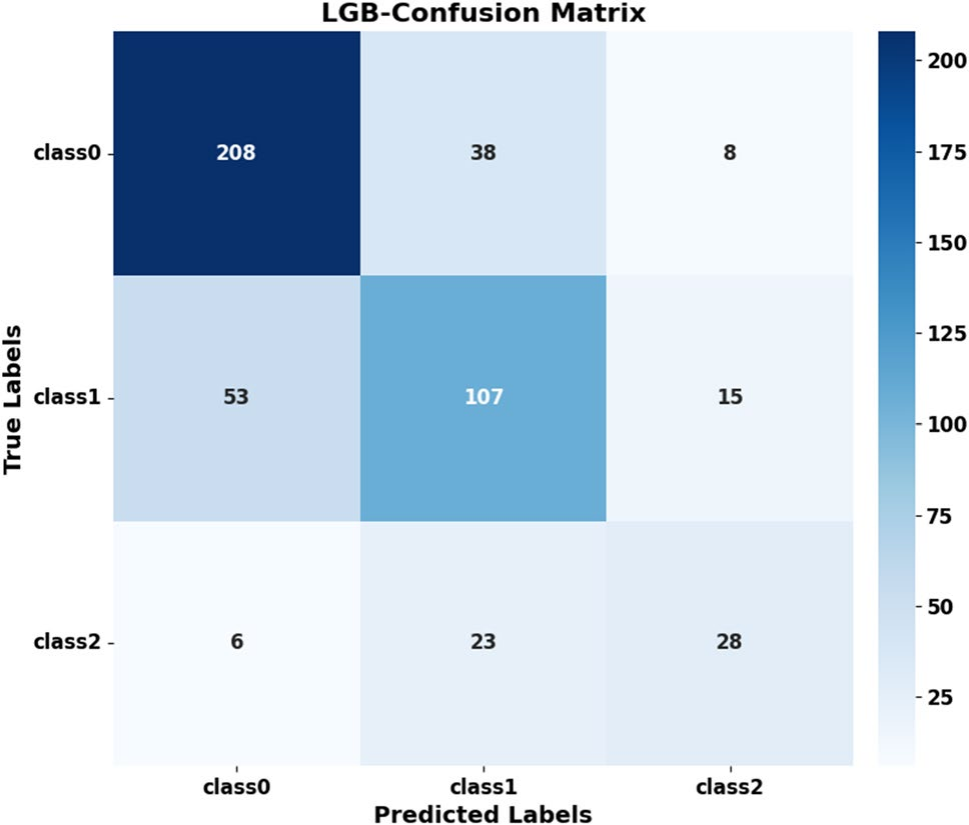
The confusion matrix of the LGB model for the test set. The y-axes class 0, class 1, and class 2 correspond to the actual number of normal blood pressure, prehypertension, and hypertension, respectively, and the x-axes class 0, class 1, and class 2 correspond to the predicted number of LGB models, respectively.

To further understand the prediction of different categories by the best model, we analyzed the confusion matrix of LGB (Fig. 4). Among the 254 people with normal blood pressure, 81.89% were correctly classified, 14.96% were wrongly classified as prehypertension, and 3.15% were wrongly classified as hypertension. Among the 175 people with prehypertension, 61.14% were correctly classified, 30.29% were wrongly classified as normal blood pressure, and 8.57% were wrongly classified as hypertension. In the 57 people with hypertension, 10.53% were wrongly classified as normal blood pressure, and 40.35% were wrongly classified as prehypertension.

### Feature importance

**Figure 5:**
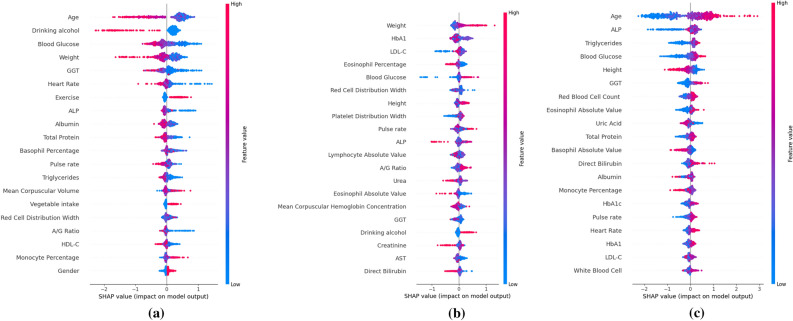
The importance of features for categories of normal blood pressure (**a**), prehypertension (**b**), and hypertension (**c**).

SHAP, developed from game theory, can be used to interpret the prediction results of various ML models^37,38^. By inputting a trained ML model into SHAP, it can generate SHAP values that reflect the contribution of each feature to the model's output. As shown in Fig. 5, age is the most critical predictive feature for normal blood pressure and hypertension, and high age values have a negative impact on the prediction result as normal blood pressure and have a positive effect on the prediction result as hypertension. No drinking habit and low blood sugar contribute positively to the prediction result as normal blood pressure, ALP and Triglycerides low values contribute negatively to the prediction result as hypertension. Weight is the most important feature for prehypertension, with high weight values having a positive impact on the prediction result as prehypertension. In addition to these, other features such as HbA1, LDL-C, and Eosinophil Percentage are also important prediction features for prehypertension.

### Risk prediction system

**Figure 6:**
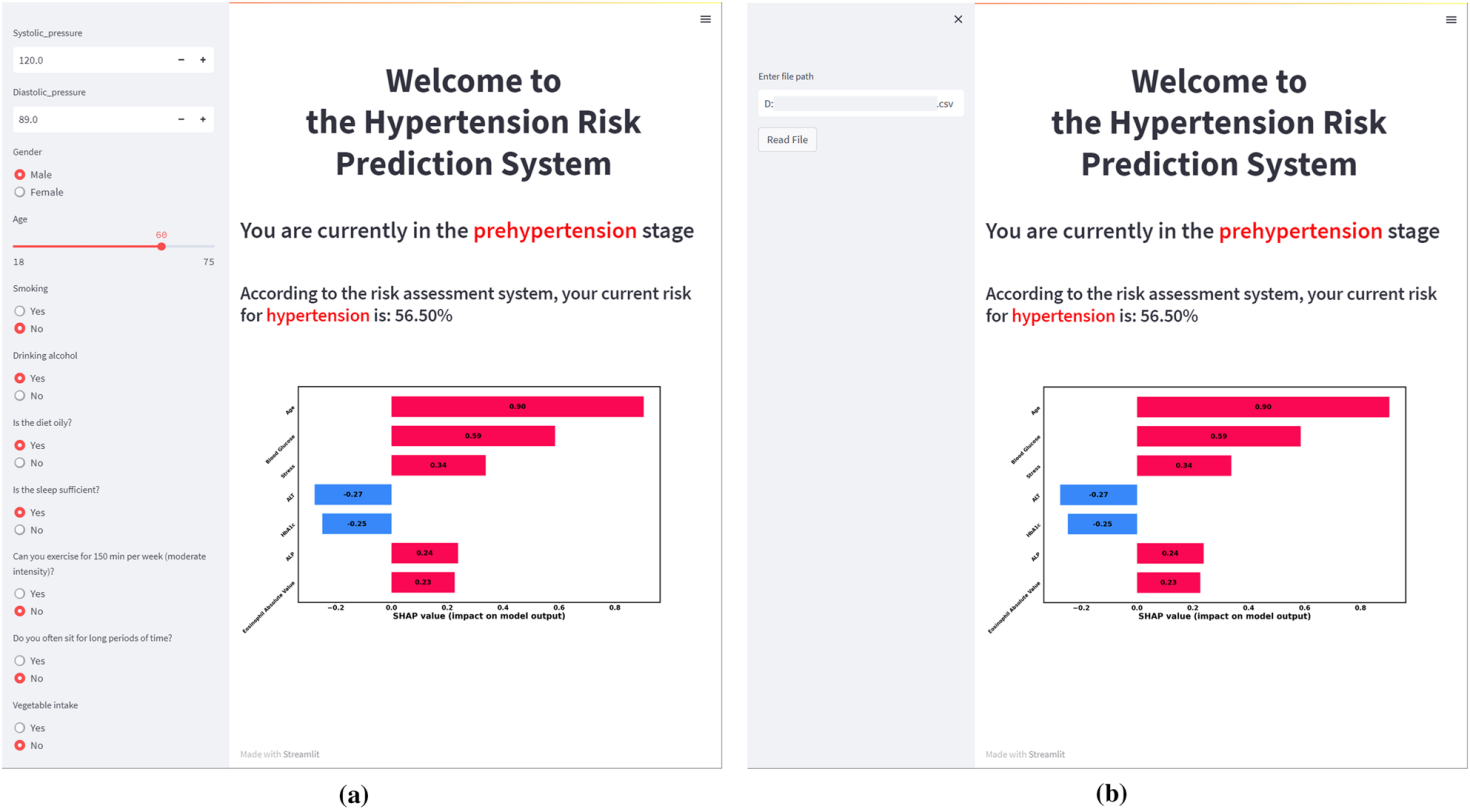
Hypertension visual risk prediction system. (**a**) The use of predicted systems by entering information one by one; (**b**) The use of predicted systems by importing information files.

Establishing a personalized health risk prediction system can help individuals and doctors better understand their health status and take appropriate preventive measures in a timely manner^39–41^. In this study, based on the interpretation of ML model output results by SHAP, a visualized hypertension risk prediction system was constructed on a webpage. As shown in Fig. 6, the prediction system is divided into two areas, with the left as the information input area and the right as the result display area. In the left area, information can be entered one by one (Fig. 6A) or by importing files (Fig. 6B). In the right area, the upper half will show the patient's current blood pressure level and the risk probability of becoming the next level, and the lower half will show the contribution of the feature variables to the risk probability, which allows doctors to devise intervention or prevention strategies.

## Discussion

This study combined ML models and the SHAP algorithm to build a visualization risk prediction system for hypertension on the web. The LGB model demonstrated the highest accuracy, average F1-score, average AUC values, proving its superiority in hypertension classification prediction. This is possibly because LGB is a feature selection method based on gradient boosting trees, and traditional models like LR are more suitable for dealing with simple data where variables and results have a linear relationship, while deep learning models like BPNN are more suitable for processing image data or natural language problems^42,43^.

Compared to similar studies that only divide the population into two categories^44^, normotensive and hypertensive, our three-category study is more comprehensive and detailed, predicting the likelihood of prehypertension in individuals with normal blood pressure, which also aids in preventing the occurrence of hypertension and reducing the risk of cardiovascular disease. At the same time, the AUC value of the ROC curve of the prediction model in this study is higher than that of similar studies (0.82 vs 0.77), showing better predictive performance^45^. Through the SHAP algorithm, we revealed the influence of different feature variables on the prediction results of hypertension. Age, ALP, triglycerides, and blood sugar are important prediction factors for hypertension. The increase in hypertension risk related to age is a result of the ageing process of the organ system^46^. Khalili et al. mentioned that ALP can catalyze the hydrolysis of the phosphatase group in the vascular wall, leading to vascular calcification, damaging vascular homeostasis, and accelerating the development of hypertension^47^. Laaksonen et al. believe that lipoproteins rich in triglycerides are toxic to endothelial cells, long-term damage to endothelial cells may lead to increased peripheral vessel resistance, resulting in higher arterial blood pressure^48^. These research results have confirmed the reliability of SHAP algorithms in interpreting the outputs of ML models. These research findings indicate that the risk factors for hypertension obtained through the SHAP algorithm are reliable.

It is worth mentioning that this study builds the prediction system on a webpage, which is the first in the research of hypertension risk prediction based on machine learning technology, which helps to enhance the practicality and user-friendliness of the prediction system. Furthermore, after inputting individual's health examination data into the prediction system, the system can rank the importance by calculating the SHAP values of each feature, providing users with an intuitive explanation of individual disease risk. As shown in Fig. 6, we demonstrated the usage of the prediction system through examples. After entering information one by one or by importing files, the system show that the current blood pressure has reached prehypertension, and the risk probability of developing into hypertension is 56.5%. Features such as age, blood glucose, pressure, ALP, and Eosinophil Percentage etc. have a positive impact on the risk probability, and ALT and HbA1c have a negative impact on the risk probability. These interpretative results can provide important guidance for doctors and patients, helping them understand the key factors affecting hypertension risk and to devise more individualized prevention and intervention strategies accordingly.

While this research has made some progress in hypertension risk prediction and enhanced the interpretability of the model by introducing the SHAP algorithm, there still exist some limitations. First of all, although we have randomly divided the dataset, single data source may still affect the generalization ability of the prediction system. At the same time, as hypertension involves many complex biological and genetic factors, future work should consider adding information such as time factors, biomarkers, and genetic factors to the risk prediction system. Finally, the prediction system built on the web is accessible only on Windows, and further optimization is needed to accommodate different operating systems.

This research successfully built a visualization risk prediction system for hypertension, using ML models and the SHAP algorithm to improve the accuracy and interpretability of the system. Building the risk prediction model on a webpage is a major innovation of this study. It helps doctors and patients to more intuitively and conveniently understand the risk probability of hypertension and the main influencing factors, and to prevent and intervene hypertension in a targeted manner. All in all, the risk prediction system we developed is accurate, reliable, practical and can be used as an effective health management tool for hypertension.

### Supplementary Information

Supplementary Information.

## Data Availability

The dataset generated and/or analyzed during the current study are not publicly available but are available from the corresponding author and the first author on reasonable request. The code was based on Python3.11 programming language (http://www.python.org). The codes are available from the corresponding author on reasonable request.
