# Supplementary material for: Developing a hypertension visualization risk prediction system utilizing machine learning and health check-up data
Source: Sci Rep. 2023 Nov 2;13:18953. doi: 10.1038/s41598-023-46281-y (PMC10622553; doi:10.1038/s41598-023-46281-y)
Supplement: Supplementary file 1 — Supplementary Information. [file 41598_2023_46281_MOESM1_ESM.docx]

**Supplementary material**

**Evaluation indicators**

$Accuracy=\frac{True Positives+True Negatives}{Total Number of Samples}$ **(Formula S1)**

$Precision=\frac{True Positives}{True Positives+False Positives}$ **(Formula S2)**

$Recall=\frac{True Positives}{True Positives+False Negatives}$ **(Formula S3)**

$F1-score=\frac{2\times Precision\times Recall}{Precision+Recall}$  **(Formula S4)**

Accuracy refers to the proportion of the number of samples correctly predicted by the model to the total number of samples. Therefore, accuracy is a global evaluation index, which does not distinguish between different categories, but considers the overall performance of the model on all categories. In contrast, precision, recall, and F1-score are indexes calculated separately for each category, so the index values of each category may be different. In this article, the average values of precision, recall, and F1-score refer to adding up the index values of these three categories and then dividing by three to get an overall evaluation.

**Hyperparameters of the best model**

The best model for this study is LGB, and its hyperparameters and candidate values are:

'num_leaves': [31, 64, 128, 256],

'max_depth': [2, 4, 8, 12, 16],

'learning_rate': [0.1, 0.01, 0.001, 0.0001],

'min_child_samples': [5, 10, 20, 50, 100],

'subsample': [0.1, 0.5, 0.8, 1.0],

'colsample_bytree': [0.1, 0.5, 0.8, 1.0]

Through 10-fold cross-validation grid search, we can obtain the best combination of hyperparameter values:

'num_leaves': 64

'max_depth': 8

'learning_rate': 0.1

'min_child_samples': 5

'subsample': 0.8

'colsample_bytree': 0.8

Among them, 'num_leaves': this hyperparameter controls the maximum number of leaf nodes in each tree. 'max_depth': this hyperparameter controls the maximum depth of each tree, that is, the number of layers in the tree. 'learning_rate': the learning rate is a hyperparameter used to control the magnitude of weight updates in each iteration in the gradient boosting algorithm. It is related to the convergence speed of iterations. 'min_child_samples': this hyperparameter controls the minimum number of samples on each leaf node to prevent overfitting. 'subsample': this hyperparameter controls the proportion of training samples used in each tree training. 'colsample_bytree': this hyperparameter controls the proportion of features used during the training process in each tree.

**Table S1**

**Missing rate, Mean, Median, and Standard Deviation of feature variables.**

|  | Missing Rate | Mean | | Median | | Standard Deviation | |
| --- | --- | --- | --- | --- | --- | --- | --- |
|  |  | Unfilled | Filled | Unfilled | Filled | Unfilled | Filled |
| Mean Platelet Volume | 0.8% | 10.749 | 10.796 | 10.6 | 10.6 | 1.014 | 1.109 |
| Plateletcrit | 0.8% | 0.256 | 0.257 | 0.25 | 0.25 | 0.053 | 0.053 |
| Platelet Distribution Width | 0.8% | 13.211 | 13.316 | 12.7 | 12.8 | 2.484 | 2.652 |
| HbA1c | 21.4% | 5.686 | 5.691 | 5.6 | 5.6 | 0.756 | 0.771 |
| HbA1 | 21.4% | 6.88 | 6.882 | 6.7 | 6.7 | 0.846 | 0.86 |
| Direct Bilirubin | 0.9% | 2.608 | 2.623 | 2.5 | 2.5 | 1.018 | 1.073 |
| Indirect Bilirubin | 0.9% | 12.482 | 12.56 | 11.4 | 11.4 | 5.22 | 5.43 |
| AST | 13.1% | 24.674 | 24.753 | 22 | 22 | 10.888 | 11.197 |
| ALT | 0.9% | 76.299 | 76.514 | 74 | 74 | 21.684 | 21.911 |
| Cohen's d | / | 0.003 | | 0 | | 0.017 | |
| *P* | / | 0.008 | | 0.317 | | 0.012 | |

**Table S2**

**Baseline clinical characteristics of different groups.**

|  | Class 0  (n=867,53.6%) | Class 1  (n=557,34.5%) | Class 2  (n=193,11.9%) | *χ*^2^*/F* | *P* |
| --- | --- | --- | --- | --- | --- |
| Gender |  |  |  |  |  |
| Male | 463 | 362 | 118 | 19.454 | 0.000 |
| Female | 404 | 195 | 75 |  |  |
| Age | 37 | 41 | 51 | 109.438 | 0.000 |
| Smoking |  |  |  |  |  |
| Yes | 66 | 121 | 45 | 69.294 | 8.974 |
| No | 801 | 436 | 148 |  |  |
| Drinking alcohol |  |  |  |  |  |
| Yes | 61 | 135 | 60 | 113.582 | 2.167 |
| No | 806 | 422 | 133 |  |  |
| Is the diet oily? (Diet) |  |  |  |  |  |
| Yes | 187 | 195 | 103 | 86.199 | 1.914 |
| No | 680 | 362 | 90 |  |  |
| Is the sleep sufficient? (Sleep) |  |  |  |  |  |
| Yes | 246 | 179 | 75 | 8.714 | 0.013 |
| No | 621 | 378 | 118 |  |  |
| Can you exercise for 150minutes per week (moderate intensity)? (Exercise) |  |  |  |  |  |
| Yes | 217 | 114 | 32 | 8.39 | 0.015 |
| No | 650 | 443 | 161 |  |  |
| Do you often sit for long periods of time? (Sedentary behavior) |  |  |  |  |  |
| Yes | 436 | 309 | 113 | 6.314 | 0.043 |
| No | 431 | 248 | 80 |  |  |
| Is your vegetable intake sufficient? (Vegetable intake) |  |  |  |  |  |
| Yes | 188 | 118 | 43 | 0.113 | 0.945 |
| No | 679 | 439 | 150 |  |  |
| Do you often feel stressed? (Stress) |  |  |  |  |  |
| Yes | 329 | 192 | 52 | 8.701 | 0.013 |
| No | 538 | 365 | 141 |  |  |
| Pulse rate (times/min) | 78 | 80 | 79 | 3.767 | 0.023 |
| Height (cm) | 166.6 | 167.8 | 164.6 | 9.495 | 0.000 |
| Weight (kg) | 64.9 | 71.4 | 71.3 | 51.72 | 0.000 |
| White Blood Cell (10^9^/L) | 6 | 6.1 | 6.2 | 1.71 | 0.181 |
| Neutrophil Percentage (%) | 56 | 56.7 | 57 | 2.107 | 0.122 |
| Lymphocyte Percentage (%) | 34.2 | 33.7 | 33.3 | 1.363 | 0.256 |
| Monocyte Percentage (%) | 7 | 6.8 | 6.7 | 2.398 | 0.091 |
| Eosinophil Percentage (%) | 2.3 | 2.1 | 2.4 | 2.842 | 0.059 |
| Basophil Percentage (%) | 0.57 | 0.57 | 0.56 | 0.147 | 0.863 |
| Neutrophil Absolute Value (10^9^/L) | 3.4 | 3.5 | 3.6 | 2.528 | 0.080 |
| Lymphocyte Absolute Value (10^9^/L) | 2.02 | 2.03 | 2.03 | 0.08 | 0.923 |
| Monocyte Absolute Value (10^9^/L) | 0.414 | 0.416 | 0.413 | 0.057 | 0.945 |
| Eosinophil Absolute Value (10^9^/L) | 0.141 | 0.133 | 0.152 | 1.951 | 0.143 |
| Basophil Absolute Value (10^9^/L) | 0.034 | 0.035 | 0.034 | 0.432 | 0.649 |
| Red Blood Cell Count (10^12^/L) | 4.7 | 4.9 | 4.9 | 18.349 | 0.000 |
| Hemoglobin (g/L) | 141.4 | 146.5 | 147.1 | 20.091 | 0.000 |
| Hematocrit (%) | 42.9 | 44.3 | 44.5 | 22.404 | 0.000 |
| Mean Corpuscular Volume (fl) | 90.6 | 90.5 | 90.9 | 0.366 | 0.694 |
| Mean Corpuscular Hemoglobin (pg) | 29.8 | 29.9 | 30 | 0.682 | 0.506 |
| Mean Corpuscular Hemoglobin Concentration (g/L) | 329 | 330 | 330.2 | 1.77 | 0.171 |
| Red Cell Distribution Width (%) | 12.6 | 12.57 | 12.62 | 0.189 | 0.828 |
| Platelet Count (10^9^/L) | 240.4 | 243.8 | 236.3 | 1.389 | 0.250 |
| Mean Platelet Volume (fl) | 10.8 | 10.78 | 10.84 | 0.226 | 0.798 |
| Plateletcrit (%) | 0.26 | 0.26 | 0.25 | 1.534 | 0.216 |
| Platelet Distribution Width (%) | 13.26 | 13.34 | 13.53 | 0.844 | 0.430 |
| HbA1c (%) | 5.58 | 5.71 | 6.15 | 45.023 | 0.000 |
| HbA1 (%) | 6.76 | 6.9 | 7.4 | 46.195 | 0.000 |
| Total Bilirubin (μmol/L) | 15.03 | 15.26 | 15.21 | 0.251 | 0.778 |
| Direct Bilirubin (μmol/L) | 2.66 | 2.58 | 2.6 | 0.936 | 0.392 |
| Indirect Bilirubin (μmol/L) | 12.4 | 12.7 | 12.8 | 0.485 | 0.616 |
| Total Protein (g/L) | 71.6 | 72.5 | 72.5 | 12.958 | 0.000 |
| Albumin (g/L) | 44.3 | 45 | 44.6 | 13.349 | 0.000 |
| Globulin (g/L) | 27.3 | 27.5 | 27.9 | 3.673 | 0.026 |
| A/G Ratio (%) | 1.65 | 1.66 | 1.62 | 2.648 | 0.071 |
| ALT (U/L) | 24.7 | 30.1 | 31 | 12.431 | 0.000 |
| AST (U/L) | 23.5 | 26 | 27 | 12.999 | 0.000 |
| ALP (U/L) | 73.6 | 78.5 | 83.7 | 20.841 | 0.000 |
| GGT (U/L) | 28.4 | 35.8 | 41.2 | 11.219 | 0.000 |
| Urea (mmol/L) | 4.8 | 5 | 5.2 | 10.413 | 0.000 |
| Creatinine (μmol/L) | 69.7 | 72.1 | 70.6 | 4.276 | 0.014 |
| Uric Acid (μmol/L) | 339.3 | 370 | 367.2 | 21.119 | 0.000 |
| Blood Glucose (mmol/L) | 5 | 5.3 | 6.1 | 54.983 | 0.000 |
| Total Cholesterol (mmol/L) | 4.7 | 5 | 5.2 | 25.202 | 0.000 |
| Triglycerides (mmol/L) | 1.5 | 1.8 | 2 | 13.306 | 0.000 |
| HDL-C (mmol/L) | 1.3 | 1.26 | 1.27 | 3.87 | 0.021 |
| LDL-C (mmol/L) | 2.78 | 2.97 | 3.13 | 29.617 | 0.000 |
| Heart Rate (times/min) | 68 | 71 | 72 | 14.594 | 0.000 |

**Table S3**

**The Precision, Recall and F1-score for ten machine learning models.**

|  | Accuracy | Precision | | | | Recall | | | | F1-score | | | |
| --- | --- | --- | --- | --- | --- | --- | --- | --- | --- | --- | --- | --- | --- |
|  |  | Average | Class 0 | Class 1 | Class 2 | Average | Class 0 | Class 1 | Class 2 | Average | Class 0 | Class 1 | Class 2 |
| RF | 0.6584 | 0.5875 | 0.8000 | 0.6131 | 0.3494 | 0.6125 | 0.7402 | 0.5886 | 0.5088 | 0.5946 | 0.7689 | 0.6006 | 0.4143 |
| XGB | 0.7016 | 0.6244 | 0.7228 | 0.5912 | 0.5593 | 0.6482 | 0.8255 | 0.5192 | 0.6000 | 0.6359 | 0.8035 | 0.5529 | 0.5789 |
| SVM | 0.5514 | 0.4905 | 0.6898 | 0.4671 | 0.3146 | 0.5208 | 0.6654 | 0.4057 | 0.4912 | 0.4984 | 0.6774 | 0.4343 | 0.3835 |
| LGB | 0.7057 | 0.6550 | 0.7790 | 0.6369 | 0.5490 | 0.6405 | 0.8189 | 0.6114 | 0.4912 | 0.6470 | 0.7985 | 0.6239 | 0.5185 |
| DT | 0.5576 | 0.5066 | 0.7170 | 0.5170 | 0.2857 | 0.5365 | 0.5984 | 0.5200 | 0.4912 | 0.5107 | 0.6524 | 0.5185 | 0.3613 |
| GBT | 0.6728 | 0.6277 | 0.7391 | 0.5941 | 0.5500 | 0.5887 | 0.8031 | 0.5771 | 0.3860 | 0.6030 | 0.7698 | 0.5855 | 0.4536 |
| MLP | 0.5967 | 0.5377 | 0.7038 | 0.5725 | 0.3368 | 0.5702 | 0.7205 | 0.4286 | 0.5614 | 0.5411 | 0.7121 | 0.4902 | 0.4211 |
| KNN | 0.5761 | 0.5087 | 0.7252 | 0.5480 | 0.2529 | 0.5247 | 0.6339 | 0.5543 | 0.3860 | 0.5111 | 0.6765 | 0.5511 | 0.3056 |
| BPNN | 0.6111 | 0.5594 | 0.7799 | 0.5543 | 0.3441 | 0.5953 | 0.6417 | 0.5829 | 0.5614 | 0.5663 | 0.7041 | 0.5682 | 0.4267 |
| LR | 0.5535 | 0.4211 | 0.6444 | 0.4188 | 0.2000 | 0.4183 | 0.8346 | 0.2800 | 0.1404 | 0.4093 | 0.7273 | 0.3356 | 0.1649 |
